# Narrative Exposure Therapy in Patients With Posttraumatic Stress Disorder and Borderline Personality Disorder in a Naturalistic Residential Setting: A Randomized Controlled Trial

**DOI:** 10.3389/fpsyt.2021.765348

**Published:** 2021-11-26

**Authors:** Carolin Steuwe, Michaela Berg, Thomas Beblo, Martin Driessen

**Affiliations:** Department of Psychiatry and Psychotherapy, Evangelisches Klinikum Bethel, Universitätsklinikum OWL of Bielefeld University, Bielefeld, Germany

**Keywords:** borderline personality disorder (BPD), narrative exposure therapy (NET), posttraumatic stress disorder (PTSD), randomized controlled trial (RCT), cognitive behavior therapy (CBT)

## Abstract

**Background:** Comorbid Posttraumatic Stress Disorder (PTSD) increases the already high symptom burden of patients with Borderline Personality Disorder (BPD). As the gold standard for BPD treatment, Dialectical Behavior Therapy (DBT), does not focus on PTSD, other treatment approaches are needed. Narrative Exposure Therapy (NET) was designed to address multiple traumatic events and may be especially useful in this patient group. The aim of the present study was to determine the efficacy of NET compared to DBT based treatment (DBT-bt) in a randomized controlled trial.

**Methods:** Female patients (*n* = 60) with BPD and comorbid PTSD were randomized to either a 10-week residential NET or DBT-bt. The primary outcome was change in PTSD severity as assessed by the Clinician Administered PTSD Scale (CAPS). Mixed linear models as well as reliable change, remission, and response rates were used to compare improvement across treatment groups.

**Results:** Mixed linear model showed that patients in both treatments improved significantly over time across all outcome measures. This improvement was not more pronounced in NET (no significant time × type of treatment effect). However, NET resulted in a higher remission rate as compared to DBT-bt. PTSD remission was accompanied by BPD remission in all cases.

**Conclusions:** This study shows the value of trauma-focused therapy in patients with BPD and PTSD for recovery in both disorders. To shorten the duration of both illnesses as much as possible, future studies should focus on the factors predicting treatment success and enabling patients to benefit from trauma-focused treatment as soon as possible.

**Trial registration:** ClinicalTrials.gov, identifier: NCT02517723.

## Introduction

About half of all patients with borderline personality disorder (BPD) suffer from comorbid posttraumatic stress disorder [PTSD; (1)]. Comorbid PTSD increases the already high symptom load by increasing emotion regulation problems, the frequency of suicide attempts, and non-suicidal self-injury (2). For the treatment of PTSD, exposure-based cognitive-behavioral treatment programs are most effective (3). However, for a long time there were reservations about the safety of exposure therapy in patient groups that exhibit high levels of dysfunctional behaviors, such as BPD (4). At the same time, Dialectical Behavior Therapy [DBT; (5)], the treatment program that has demonstrated its effectiveness in BPD most often, was not found to be sufficiently effective on PTSD symptomatology in patients with BPD and PTSD (6). In order to improve treatment for this seriously ill patient group, trauma exposure has been applied to patients with BPD despite the safety concerns in recent years.

Three randomized-controlled trials by Harned et al. (7) and Bohus et al. (8, 9) showed outstanding results in decreasing PTSD as well as BPD symptom severity in both outpatient and residential settings. Both research groups use a phase-based approach applying DBT before complementing treatment with a trauma-focused cognitive-behavioral intervention or prolonged exposure to ensure safety and in order to treat both PTSD and BPD related symptoms. Harned et al. (7) compared DBT with or without Prolonged Exposure (PE) in an outpatient setting and showed that patients who received DBT+DBT-PE experienced a significantly greater reduction in PTSD symptoms than patients who received DBT alone. A reanalysis of their data also showed that changes in comorbid conditions did not occur until PTSD was successfully treated (10). Bohus et al. (8) integrated DBT and an exposure procedure into an overall treatment program (DBT-PTSD). In a residential setting, it was compared to a treatment-as-usual wait list. DBT-PTSD led to a significantly higher reduction in PTSD symptom burden while showing a low treatment dropout rate. The results were confirmed in a large outpatient study by the same research group (9). DBT-PTSD proved to be superior in reducing PTSD severity and dropout rates compared to Cognitive-Processing-Therapy [CPT; (11)]. Although most studies used a 3-months follow-up interval and long-term effects still need to be shown, findings indicate that there should be good reasons not to offer trauma-focused treatment in comorbid PTSD and BPD. New research questions arise from this advance in treatment. For example, there is little evidence to date comparing trauma-focused treatment with standard BPD treatment (DBT). Also, the choice of the trauma-focused treatment method is unclear, especially with regard to the necessity of a phase-based approach. Our goal is to expand the existing evidence base with respect to these points, taking into account long-term effects.

Narrative exposure therapy [NET; (12)] aims to combine highly emotional trauma memories with the correct situational and temporal contextual information promoting a coherent autobiographical memory associated with sensory, affective, and cognitive features of the experience (13). It was specifically designed and proved to be effective for patients who have experienced multiple events and different types of traumatic experiences (14). As BPD patients with PTSD commonly experience many traumatic events (15), NET could be advantageous. In two pilot studies, NET was feasible, safe and potentially effective in female patients with BPD and PTSD (16, 17). However, randomized-controlled trials are required to further evaluate NET in this patient group.

In this paper, we conducted an evidence-based trauma therapeutic treatment using a non-phase-based approach, Narrative Exposure Therapy, on patients with PTSD plus BPD in a residential setting. We aimed to evaluate NET in severely ill patients with both BPD and PTSD on the recovery of primarily PTSD but also BPD compared to DBT based treatment in a naturalistic residential setting including a 12-months follow-up. We hypothesize that NET is superior to DBT-bt at 12-months follow-up with regard to reductions in PTSD symptom severity evident in (a) a pronounced symptom reduction over time and (b) in higher numbers in categorical measures of change, e.g., remission rates.

## Methods

### Study Site and Study Design

This single-site study was conducted at the ward for patients with BPD at the Clinic of Psychiatry and Psychotherapy, Ev. Klinikum Bethel, Universitätsklinikum OWL, Bielefeld University, Germany. We conducted a randomized controlled trial comparing NET with standard DBT (DBT based treatment (DBT-bt), for explanation see Interventions section) both integrated in a residential setting. After completion of assessment procedures, eligible participants were randomly assigned in a 1:1 ratio to either NET or DBT-bt. Patients were examined pre- and post-treatment and 12 months after discharge. The periods of enrollment and follow-up ran from July 2013 to January 2020. The study was approved by the University of Muenster Ethics Committee (date of approval: July 8th 2013) and is in accordance with the Declaration of Helsinki. All patients provided written consent for the study procedures.

### Participants and Procedures

Within preliminary talks for our residential treatment program, patients were consecutively screened for the inclusion criteria. In case of positive screening, patients were invited to participate in the study and further diagnostics. Inclusion criteria included female gender, being aged 18–65 years as well as meeting a DSM-IV-TR defined diagnosis of BPD and PTSD (18). To ensure that only seriously ill patients were treated for whom there was an indication for inpatient/residential treatment, a score of at least 50 points on the Clinician-Administered PTSD Scale [CAPS; (19)] was required with regard to PTSD and at least one non-successful previous outpatient treatment with regard to BPD. Exclusion criteria were clinically evaluated doubts about the capacity to consent and to contract, pregnancy, or breastfeeding, a lifetime diagnosis of schizophrenia, body mass index <16.5, current substance use, a suicide attempt 2 months before admission, ongoing victimizing perpetrator contact, and undergoing a DBT- or exposure-based therapy within the last 12 months. Non-suicidal self-injuries were explicitly not an exclusion criterion.

### Assessments and Measures

To further assess the inclusion and exclusion criteria after positive screening, patients were invited to attend an outpatient diagnostic appointment before the start of treatment. During this appointment, the diagnostic interviews were conducted. Pretreatment assessments were conducted by independent clinical assessors who were blind to treatment condition. At post-treatment and at 12-months follow-up participants were asked to retain their treatment condition, however, this was not always successful. Within the first week after admission, i.e., after inclusion in the study and randomization, questionnaires were completed.

#### Diagnostic Interviews

The Structured Clinical Interview for DSM-IV Axis I Disorders (SCID-I) and Axis II Disorders (SCID-II) were administered by clinician raters to diagnose Axis I and Axis II disorders (20–22). The primary outcome PTSD severity was assessed via the Clinician-Administered PTSD Scale [CAPS; (19, 23)]. Notably, the CAPS was assessed with regard to all traumatic events experienced by the patient, not only one index trauma. A reliable change in PTSD symptoms was calculated as a change of ± 19.09 points on the CAPS [κ = 0.78; (24)]. BPD severity was determined by recording the number of borderline criteria fulfilled according to the SCID-II interview before treatment and at 12-months follow-up.

#### Questionnaires

The Posttraumatic Stress Diagnostic Scale [PDS; (25, 26)] was used to capture PTSD severity. Further secondary outcomes were scores on standard self-report measures of psychopathology and social functioning: the Dissociative Experiences Scale [DES; (27, 28)], the Beck Depression Inventory [BDI-II; (29, 30)], the Symptom Checklist-90-Revised [SCL-90-R; (31, 32)], and the brief version of the Quality of Life questionnaire of the World Health Organization [WHOQOL; (33)].

### Interventions

Both interventions were integrated in a residential treatment program, which are common in many European countries [e.g., (34)] to treat patients who have not benefitted from outpatient treatment or are not able to receive the treatment needed, for example because of safety concerns. Both treatments lasted 10 weeks. For information on standard inpatient care and for further description of therapy dose and therapists, see Supplementary Material.

#### Narrative Exposure Therapy

The treatment followed the protocol described in the pre-ceding pilot study (17). During the first sessions, patients received psychoeducation on trauma, PTSD and the NET procedures. They revisited and practiced techniques to interrupt dissociation and reduce tension. Furthermore, patients created an individual lifeline with positive and negative/traumatic life events. Afterwards, patients received 12 sessions (session 4–15) of trauma exposure via NET. In the last two sessions, patients received their narrative and short cognitive interventions, as needed, to reduce emotions of shame and guilt. Patients were also encouraged to reintegrate into and refocus on their daily routines. Weekly sessions of 50 min occurred before and after the exposure phase. During the exposure period (beginning in week 3) individual sessions of 90 min occurred twice a week.

#### Dialectical Behavior Therapy

DBT is a cognitive-behavioral treatment program that was developed to treat suicidal patients with BPD (5). Participants received weekly 50-min sessions of individual treatment (ten sessions over the 10 weeks) plus the following weekly group treatments: 180 min of skills training, 45 min of practicing mindfulness and psychoeducation on BPS as well as 60 min of patient-guided support group (ten sessions each over the 10 weeks). DBT aims to help patients achieve the following: (1) reduction of suicidal behaviors, (2) reduction of therapy-interfering behaviors, and (3) other risky or destabilizing behaviors. Standard DBT aims to achieve these goals by (1) conveying behavioral capabilities (skills), (2) motivating to apply these skills, (3) helping to generalize learned skills to the patient's natural environment, (4) structuring the treatment environment to reinforce functional behavior, and (5) conveying therapeutic resources and motivation to effectively treat patients with BPD.

The ward is a DBT treatment unit certified by the DBT roofing association in Germany (DDBT). During the study procedure the treatment unit was consecutively certified from 2007 to 2016 and from 2018 to date (certification takes place every 2 years). The certification pause arose because certified employees left the ward unexpectedly and the required number of certified employees was no longer met. The station was recertified as soon as possible. Since the ward was not certified for the entire study period, we refer to it as DBT based treatment (DBT-bt).

### Statistical Analysis

All analyses were conducted on the treatment completer sample (TC) and the intention-to-treat sample (ITT). To avoid a bias, we included all participants in our ITT-analyses that participated in at least one diagnostic interview, even if they refused to complete questionnaires pre- or post-treatment or at 12-months follow-up (see Supplementary Table 1 in the Supplementary Material). Mixed linear models with random slopes and intercepts were used to test whether decrease in symptom severity over time was more pronounced under NET compared to the DBT-bt group (group × time interaction). Besides the interaction term, all models included the main effects of time (three time points, two time points for SCID-II-BPD criteria as they were only covered pre-treatment and at follow-up) and treatment condition (NET vs. DBT-bt) as well as variables in which there were significant differences between the groups at the pretime point. All variables were checked for violation of model assumptions by residual analyses. Individual missing values (<10%) were imputed separately for both treatment groups using predictive mean matching based on all other items from the respective scale (25 imputations). SPSS 25 (35) was used to conduct multiple imputations. Treatment estimates were combined using Rubin's rules (36). Mixed effects models [package nlme; (37)] were carried out in R using RStudio (38). Beyond mixed linear models that were carried out on all outcome variables, categorical change measures were analyzed. PTSD treatment response was defined as a reduction of at least 30 points in the CAPS score (39), beyond that the reliable change index was calculated (40). PTSD-remission was defined as not meeting DSM-IV PTSD criteria any longer according to the CAPS using the “1–2”-rule [frequency score of at least one and intensity score of at least two; (19)]. BPD-remission was defined as meeting less than five DSM-IV BPD-criteria according to the SCID-II interview. Continuous data were compared across treatment groups using *t*-tests or Mann-Whitney-*U*-tests, depending on fulfillment of assumptions. Dichotomous data were compared across treatment groups using χ^2^-tests. Within-group effect sizes were calculated using Hedges' *g*. A significance level of ≤ 0.05 (two-tailed) was used for all analyses.

## Results

### Patient Flow and Participant Characteristics

**Figure 1:**
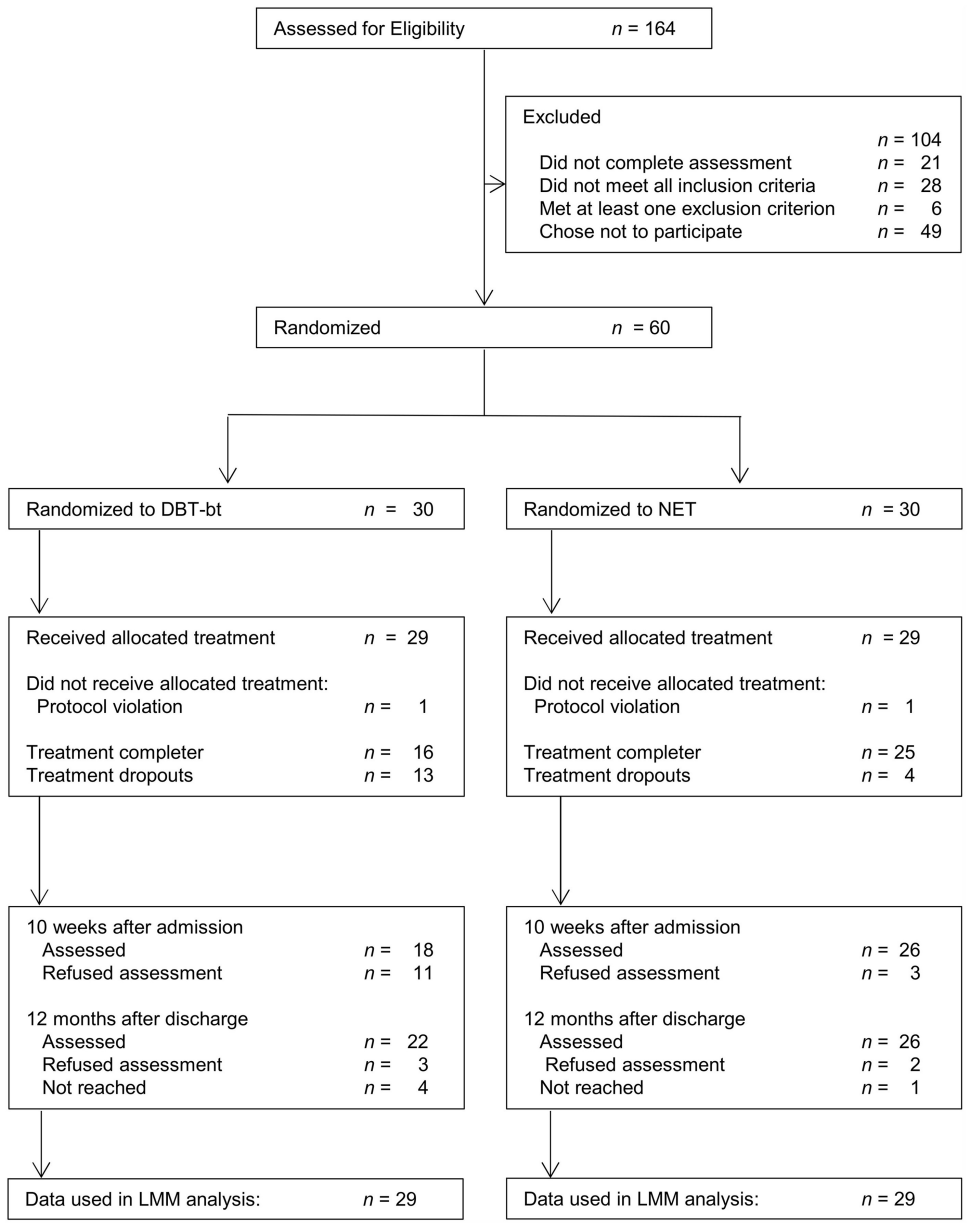
Patient flow. DBT-bt, Dialectical Behavior Therapy (DBT) based treatment; NET, Narrative Exposure Therapy; LMM, Linear Mixed Models.

One hundred sixty-four patients were assessed for study participation within the recruitment period (Figure 1). Of the 60 patients who were randomized to either NET (*n* = 30) or DBT-bt (*n* = 30), two patients (one in each group) were excluded from the study due to protocol violations (wrong treatment delivered (*n* = 1) and false information given in the diagnostic interview (*n* = 1). A total of 58 participants (NET, *n* = 29; DBT-bt, *n* = 29) started the allocated intervention (intention-to-treat sample). Five individuals randomized to NET dropped out of treatment and 13 randomized to DBT-bt dropped out of treatment. Reasons for dropout are shown in Supplementary Table 2 in Supplementary Material.

On average, participants had a mean CAPS score of 77.67 (*SD* = 14.68) and met 6.97 (*SD* = 1.30) BPD criteria. The average number of additional Axis I disorders was 1.87 (*SD* = 1.09). Most common was a major depression in 60% of all patients. The most common traumatic event was physical assault (94.7%) followed by sexual assault (82.5%) and armed attacks (45.6%). A history of one or more events of NSSI behaviors during the 4 weeks prior to admission was reported by 59.5% of participants (*median* 4.0, *range* 1–123); with a rate of 55% (*median* 2.5, *range* 1–123) in the NET group and of 64.5% (*median* 4.0, *range* 1–44) in the DBT-bt group. 82.9% of patients experienced suicidal thoughts 4 weeks before admission. All patients had attempted suicide at least once (*median* 4.0, *range* 1–76). Baseline trauma data are shown in Table 1.

**Table 1:** Patient characteristics and psychotropic medication.

	**Treatment completer sample (*****n*** **=** **40)**		**Intention-to-treat sample (*****n*** **=** **58)**	
	**NET**	**DBT-bt**	**NET**	**DBT-bt**
Age, years (*M, SD*)	31.50 (7.76)	31.88 (9.11)	30.82 (8.34)	31.27 (8.24)
Years of education (*M, SD*)	10.36 (0.95)	10.40 (0.99)	10.50 (1.07)	10.40 (1.16)
Number of Axis I disorders (*M, SD*)	2.04 (1.27)	1.88 (0.96)	1.96 (1.26)	1.71 (0.91)
PTSD severity assessed by CAPS (*M, SD*)	78.18 (11.77)	76.94 (15.68)	77.32 (11.36)	77.74 (17.36)
Number of BPD criteria (*M, SD*)	6.96 (1.23)	6.75 (1.24)	7.03 (1.27)	6.90 (1.35)
**Childhood trauma history (CTQ)**				
Sexual abuse (*M, SD*)	16.07 (7.12)	15.52 (7.93)	16.44 (6.85)	14.79 (8.36)
Physical abuse (*M, SD*)	13.36 (6.75)	13.23 (7.56)	13.37 (6.80)	11.74 (7.04)
Emotional abuse (*M, SD*)	21.04 (4.57)	20.81 (4.40)	20.52 (4.57)	19.78 (5.38)
Physical neglect (*M, SD*)	14.52 (4.65)	13.95 (5.44)	14.04 (4.94)	13.13 (5.44)
Emotional neglect (*M, SD*)	20.22 (2.65)	20.41 (4.79)	19.96 (2.67)	19.18 (5.70)
**Psychotropic medication at baseline**				
Any psychotropic medication *(n, %)*	20 (83.3)	15 (93.8)	25 (86.2)	23 (79.3)
Antidepressants *(n, %)*	15 (62.5)	14 (87.5)	18 (62.1)	20 (69.0)
Antipsychotics *(n, %)*	15 (62.5)	11 (68.8)	19 (65.5)	17 (58.6)
Mood stabilizers *(n, %)*	–	2 (12.5)	–	2 (6.9)
Stimulants (*n*, %)	–	1 (6.3)	–	1 (3.4)
Benzodiazepines *(n, %)*	–	–	–	–
Other *(n, %)*	2 (8.3)	–	3 (10.3)	–
**Psychotropic medication at follow-up**				
Any psychotropic medication *(n, %)*	11 (68.8)	13 (86.7)	14 (73.7)	15 (78.9)
Antidepressants *(n, %)*	9 (56.3)	9 (60.0)	11 (57.9)	11 (57.9)
Antipsychotics *(n, %)*	6 (37.5)	8 (53.3)	9 (47.4)	9 (47.4)
Mood stabilizers *(n, %)*	–	2 (13.3)	–	2 (10.5)
Stimulants *(n, %)*	–	–	–	–
Benzodiazepines *(n, %)*	–	–	–	1 (5.3)
Other *(n, %)*	1 (6.7)	1 (6.7)	1 (5.6)	2 (10.5)

*NET, Narrative Exposure Therapy; DBT-bt, Dialectical Behavior Therapy (DBT) based treatment (DBT-bt); CAPS, Clinician Administered PTSD Scale (19); PTSD, Posttraumatic Stress Disorder; BPD, Borderline Personality Disorder; CTQ, Childhood Trauma Questionnaire (41). There was no significant difference in group comparisons in any sample (p > 0.05)*.

NET group and DBT-bt group did not differ at baseline with regard to the main outcome scores, demographics, and most clinical measures. There was a difference between both groups with regard to the BDI (*t* = -2.96, *p* = 0.005), with higher values in the DBT-bt group at baseline. Also, the number of patients who had experienced sexual abuse differed between groups (NET: *n* = 27, DBT-bt: *n* = 21, $\chi_{\left(1\right)}^{2}$ =4.35, *p* = 0.037). Pre-treatment depression and the experience of sexual abuse were therefore included as fixed effects in the mixed-effects models.

Treatment completion rates differed between treatment conditions (NET = 82.8%, DBT-bt = 55.2%, $\chi_{\left(1\right)}^{2}$ = 5.156, *p* = 0.023). Treatment groups did not differ between groups with regard to whether the discharge was initiated by the ward (NET: 40%, DBT-bt: 54%) or by the patient herself, $\chi_{\left(1\right)}^{2}$ = 0.28, *p* = 0.599.

At 12-months follow-up there were no differences between groups with regard to critical life events and psychotherapeutic care between treatment conditions in the follow-up period. Results are presented in Supplementary Table 3 in the Supplementary Material. To our knowledge, no patient committed suicide.

### Clinical Outcomes

Results of mixed-effects models for all outcomes are shown in Table 2, descriptive data and within-group Hedge's *g* effect sizes are shown in Table 3. Pre-treatment differences in depression did not affect the results of the mixed-models presented below, however, the inclusion of experience of sexual abuse as a fixed factor lead to significant interaction effects. All analyses were therefore conducted on the total sample (each for the ITT and TC sample) as well as on the subsample of patients with sexual abuse (results did not differ, see Supplementary Material).

**Table 2:** Results of mixed-effects models for all outcome measures.

	**Treatment completer sample (*****n** **=*** **40)**			**Intent-to-treat sample (*****n** **=*** **58)**		
	**Main effects**		**Interaction**	**Main effects**		**Interaction**
	**Time**	**Treatment**	**Time × treatment**	**Time**	**Treatment**	**Time × treatment**
CAPS	21.48_(2, 64)_^***^	0.14_(1, 35)_	0.16_(2, 64)_	23.42_(2, 76)_^***^	0.04_(1, 46)_	0.04_(2, 76)_
PDS	8.00_(2, 64)_^***^	0.03_(1, 37)_	0.77_(2, 64)_	21.21_(2, 74)_^***^	0.33_(1, 46)_	0.53_(2, 74)_
SCID-II-BPD	32.34_(1, 33)_^***^	0.47_(1, 36)_	0.11_(1, 33)_	44.00_(1, 40)_^***^	<0.01_(1, 47)_	0.32_(1, 40)_
BSL	18.61_(2, 62)_^***^	0.67_(1, 35)_	1.34_(2, 62)_	24.68_(2, 74)_^***^	0.46_(1, 46)_	0.57_(2, 74)_
BDI-II	$30.59_{\left(2,66\right)}^{***}$	4.66_(1, 38)_^*^	1.33_(2, 66)_	34.61_(2, 78)_^***^	7.90_(1, 52)_^**^	0.80_(2, 78)_
DES	9.89_(2, 61)_^***^	0.09_(1, 35)_	0.89_(2, 61)_	10.81_(2, 72)_^***^	<0.01_(1, 46)_	0.48_(2, 72)_
SCL-90-R	15.74_(2, 61)_^***^	2.58_(2, 35)_	0.07_(2, 61)_	19.50_(2, 72)_^***^	1.70_(1, 46)_	0.03_(2, 72)_
WHOQOL	5.41_(2, 60)_^**^	0.02_(1, 35)_	0.53_(2, 60)_	10.52_(2, 74)_^***^	1.17_(1, 47)_	0.85_(2, 74)_

*All results are presented as F-values (F_d1, d2_). Baseline depression was entered as a fixed factor in all models except for the depression models. NET, Narrative Exposure Therapy; DBT-bt, Dialectical Behavior Therapy (DBT) based treatment; CAPS, Clinician Administered PTSD Scale (19); SCID-II, Structured Clinical Interview for DSM-IV, Personality Disorders; BPD, Borderline Personality Disorder; PDS, Posttraumatic Stress Diagnostic Scale; BSL, Borderline Symptom List; BDI-II, Beck Depression Inventory Revision; DES, Dissociative Experience Scale; SCL-90-R, Symptom Checklist 90 Items Revised Version; WHOQOL, World Health Organization Quality of Life (higher values indicate more quality of life).*

*p ≤ 0.05, **p ≤ 0.01, ***p ≤ 0.001.

**Table 3:** Outcome data at all measuring points and within-group effect sizes.

	**Treatment completer sample (*****n** **=*** **40)**		**Intent-to-treat sample (*****n** **=*** **58)**	
	**NET (*n =* 25)**	**DBT-bt (*n =* 16)**	**NET (*n =* 29)**	**DBT-bt (*n =* 25)**
**CAPS**				
Pre-treatment (*M, SD*)	78.50 (12.02)	76.94 (15.68)	77.59 (11.60)	77.74 (17.36)
Post-treatment (*M, SD*)	55.18 (24.43)	54.87 (15.78)	54.40 (23.67)	56.58 (15.63)
12-months follow-up (*M, SD*)	56.24 (31.06)	61.68 (20.67)	58.40 (32.26)	60.54 (23.58)
Hedges' *g* (pre-post)	1.2	1.4	1.3	1.3
Hedges' *g* (pre-FU)	1.0	0.8	0.8	0.8
**PDS**				
Pre-treatment (*M, SD*)	34.31 (6.69)	34.70 (10.24)	34.88 (7.00)	35.49 (10.16)
Post-treatment (*M, SD*)	27.88 (11.26)	27.45 (10.72)	26.78 (11.68)	28.65 (10.79)
12-months follow-up (*M, SD*)	23.69 (12.70)	27.10 (10.41)	24.10 (12.93)	26.92 (10.36)
Hedges' *g* (pre-post)	0.7	0.7	0.9	0.7
Hedges' *g* (pre-FU)	1.1	0.7	1.1	0.8
**SCID-II-BPD**				
Pre-treatment (*M, SD*)	6.96 (1.23)	6.75 (1.24)	7.03 (1.27)	6.90 (1.35)
Post-treatment (*M, SD*)	–	–	–	–
12-months follow-up (*M, SD*)	4.76 (2.72)	4.31 (2.27)	4.80 (2.67)	4.41 (2.13)
Hedges' *g* (pre-FU)	1.1	1.3	1.1	1.4
**BSL**				
Pre-treatment (*M, SD*)	2.08 (0.69)	2.35 (0.44)	2.13 (0.69)	2.30 (0.52)
Post-treatment (*M, SD*)	1.62 (0.72)	1.50 (0.59)	1.57 (0.73)	1.63 (0.68)
12-months follow-up (*M, SD*)	1.61 (0.82)	1.71 (0.70)	1.62 (0.84)	1.62 (0.80)
Hedges' *g* (pre-post)	0.7	1.6	0.8	1.1
Hedges' *g* (pre-FU)	0.6	1.1	0.7	1.0
**BDI-II**				
Pre-treatment (*M, SD*)	36.76 (9.44)	43.31 (6.22)	36.65 (9.00)	43.19 (6.15)
Post-treatment (*M, SD*)	25.75 (12.85)	25.95 (12.74)	25.31 (12.47)	27.70 (12.95)
12-months follow-up (*M, SD*)	24.88 (15.52)	31.17 (12.14)	25.47 (15.06)	30.17 (12.75)
Hedges' *g* (pre-post)	1.1	1.7	1.2	1.6
Hedges' *g* (pre-FU)	1.1	1.3	1.1	1.3
**DES**				
Pre-treatment (*M, SD*)	30.39 (17.41)	35.34 (18.01)	31.27 (17.42)	27.38 (20.60)
Post-treatment (*M, SD*)	25.07 (15.75)	24.94 (14.92)	24.48 (15.47)	26.77 (14.47)
12-months follow-up (*M, SD*)	19.62 (14.07)	24.00 (18.00)	21.61 (17.79)	22.21 (17.07)
Hedges' *g* (pre-post)	0.3	0.6	0.4	0.3
Hedges' *g* (pre-FU)	0.7	0.6	0.5	0.6
**SCL-90-R**				
Pre-treatment (*M, SD*)	1.92 (0.55)	2.19 (0.59)	1.94 (0.57)	2.08 (0.60)
Post-treatment (*M, SD*)	1.39 (0.72)	1.55 (0.64)	1.35 (0.72)	1.62 (0.62)
12-months follow-up (*M, SD*)	1.49 (0.74)	1.73 (0.68)	1.50 (0.78)	1.57 (0.75)
Hedges' *g* (pre-post)	0.8	1.0	0.9	0.8
Hedges' *g* (pre-FU)	0.7	0.7	0.7	0.8
**WHOQOL—Global score**				
Pre-treatment (*M, SD*)	30.68 (18.39)	28.90 (11.83)	31.50 (18.44)	25.00 (13.36)
Post-treatment (*M, SD*)	36.98 (18.97)	41.40 (19.75)	38.46 (19.66)	38.82 (19.50)
12-months follow-up (*M, SD*)	37.50 (21.41)	38.33 (21.37)	40.00 (20.92)	40.00 (20.92)
Hedges' *g* (pre-post)	-0.3	-0.7	-0.4	-0.8
Hedges' *g* (pre-FU)	-0.3	-0.6	-0.4	-0.9

NET, Narrative Exposure Therapy; DBT-bt, Dialectical Behavior Therapy; CAPS, Clinician Administered PTSD Scale (Blake, 2000; PDS, Posttraumatic Stress Diagnostic Scale; SCID-II, Structured Clinical Interview for DSM-IV, Personality Disorders; BPD, Borderline Personality Disorder; BSL, Borderline Symptom List; BDI-II, Beck Depression Inventory Revision; DES, Dissociative Experience Scale; SCL-90-R, Symptom Checklist 90 Items Revised Version; WHOQOL, World Health Organization Quality of Life (higher values indicate more quality of life). For information on significant differences between groups and across time points, see text *p ≤ 0.05, **p ≤ 0.01, ***p ≤ 0.001.

### Primary Outcome

#### Mixed Linear Models and Effect Sizes

The mixed-effects models on PTSD symptom severity assessed by CAPS showed a significant reduction across time in both TC and ITT sample. However, there was no time x treatment interaction indicating that the treatment groups did not improve differently over time (see Table 2). The time effect traced back to a significant decrease of PTSD symptom severity between pre- and post-treatment time points (*p* ≤ 001). Results remained stable until 12-months follow-up.

Effect sizes were large for pre-post changes on the CAPS score in both treatment conditions (NET: *g* = 1.2; DBT-bt: *g* = 1.3) and large in NET (*g* = 1.0) and moderate in DBT-bt (*g* = 0.8) at 12-months follow-up. See Table 3 for results of the ITT sample.

#### Reliable Change, Response, and Remission Rates

**Figure 2:**
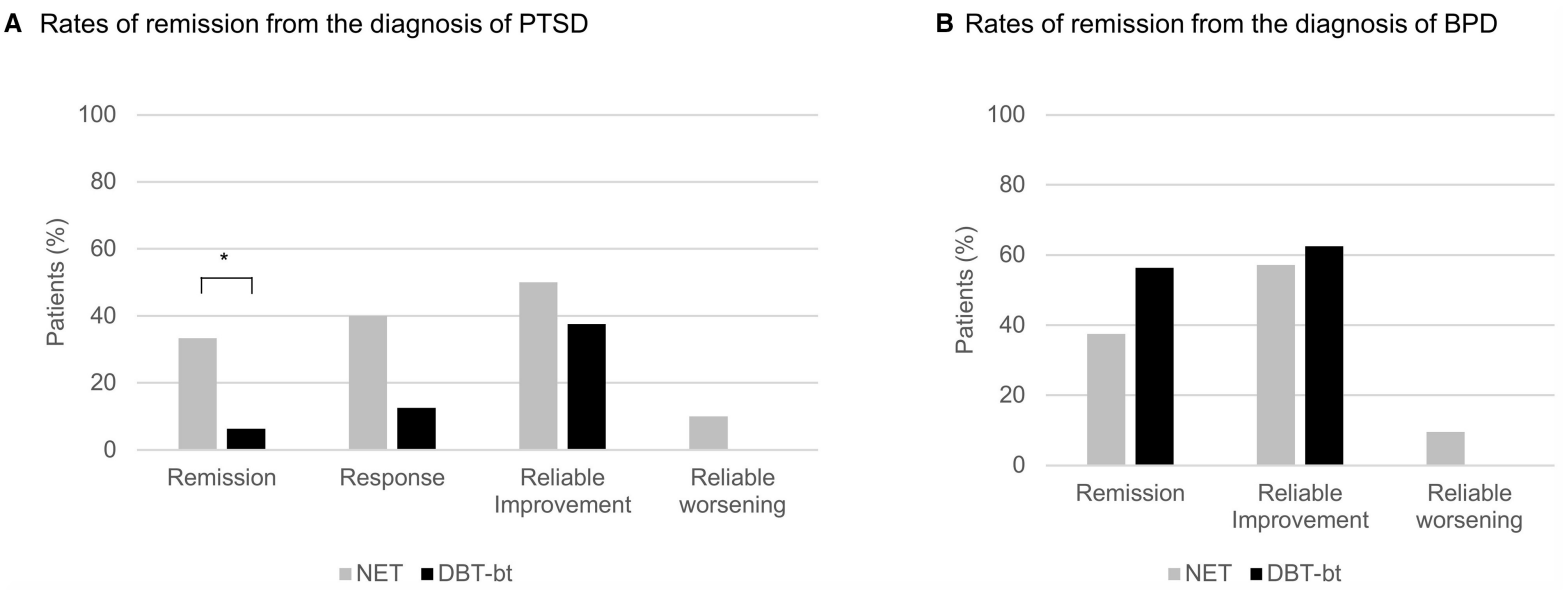
**(A)** Total Clinician-Administered PTSD Scale for DSM-IV [CAPS; (19)] scores of participants randomized to Narrative Exposure Therapy (NET; gray color) or Dialectical Behavior Therapy (DBT) based treatment (DBT-bt; black color) before therapy and at 12-months follow-up. Values below the main diagonal indicate improvements; the dotted diagonals show reliable change. **(B)** Rates of remission from the diagnosis of PTSD as well as response, reliable improvement and worsening. Data are presented for the treatment completer sample, for the intention-to-treat sample see Supplementary Figure S2 in the Supplementary Material. *p ≤ 0.05.

Comparisons of individual CAPS scores before treatment and at 12-month follow-up (Figure 2A) indicated that most participants in both groups showed improvement on the primary outcome. At 12-months follow-up, the rate of reliable change on CAPS score was slightly, but not significantly higher in NET (50%) than in DBT-bt (37.5%; see Figure 2B). No patient experienced reliable decline in the DBT-bt group, however, two patients in the NET-group had reliably worsened at 12-months follow-up compared to baseline. No patient showed reliable worsening at post-treatment. PTSD response as defined by at least 30 points reduction on CAPS at 12-months follow-up was highest in NET (TC: 40.0%, ITT: 37.5%), rates were by trend lower in DBT-bt (TC: 12.5%, ITT: 18.2%), χ^2^_(1)_ = 3.351, *p* = 0.067. This trend was also supported by the PTSD-remission rate, which was significantly higher in observed patients in NET (33.3%, ITT: 32%) than in DBT-bt (6.2%, ITT: 9.1%), χ^2^_(1)_ = 3.931, *p* = 0.047 (see Figure 2B). All of the patients who remitted from PTSD also remitted from BPD (in both NET and DBT-bt). However, only 44.4% of patients who remitted from BPD also remitted from PTSD.

It may be due to a significantly higher variance in PTSD severity at 12-months follow-up that the superiority of NET in remission rates is not also evident in the mixed models and effect sizes, *F* = 4.67, *p* = 0.043 (see also Supplementary Figure 3 in the Supplementary Material).

### Secondary Outcomes

#### Mixed Linear Models and Effect Sizes

All secondary outcomes showed a significant effect of time on symptom severity but no significant interaction effect of time and treatment (Table 2). In all models except for SCID-II-BPD (assessed only at baseline and follow-up) the significant effect of time traced back to significant pre-post changes, results remained stable until follow-up.

The effect sizes on the PDS at post-treatment were overall lower (NET and DBT-bt: *g* = 0.7) as compared to the effect sizes for the CAPS, but increased in NET to *g* = 1.1 at 12-months follow-up (DBT-bt: *g* = 0.7). There were large effect sizes for changes in Borderline criteria at 12-months follow-up in both treatment conditions (g ≥ 1.1), with the largest effect found in DBT-bt (*g* = 1.3). Effect sizes on the BSL were large in DBT-bt and moderate in NET at post-treatment (DBT-bt: *g* = 1.6, NET: *g* = 0.6) and 12-months follow up (DBT-bt: *g* = 1.1, NET: *g* = 0.7). For ITT sample, see Table 3. With regard to all other secondary outcomes, effect sizes were large with for depression and overall psychopathology; effect sizes were lower for dissociation and quality of life (Table 3).

## Discussion

In this study, we compared the efficacy of Narrative Exposure Therapy (NET) and Dialectical Behavior Therapy (DBT) based treatment (DBT-bt) for highly burdened patients with Borderline Personality Disorder (BPD) and comorbid Posttraumatic Stress Disorder (PTSD) in a randomized trial. To our knowledge, this is the first randomized study to investigate the effect of a non-phase-based trauma-focused approach compared to standard DBT based treatment in a naturalistic, residential setting with a comparably large sample size and a long follow-up interval (12 months after discharge). Our hypotheses were partially confirmed. NET showed a significantly higher remission rate than DBT-bt. Noteworthy, PTSD remission was in all cases accompanied by BPD remission. Mixed linear model showed that patients in both treatments improved significantly over time across all outcome measures. However, this improvement was not more pronounced in any treatment condition (no significant time x treatment effect).

Our results are in line with other studies showing the value of trauma-focused therapy in this highly burdened patient group (7–9, 17). The large PTSD within-group effect sizes found in our study (CAPS: *g* = 1.0, PDS: *g* = 1.1 at follow-up) are comparable to those found in two studies evaluating DBT-PTSD in a residential setting (8, 42). With respect to borderline symptom severity, both DBT-bt (SCID-II-BPD: *g* = 1.3) and NET (SCID-II-BPD: *g* = 1.1) showed large effect sizes in our study, although skills were not taught directly in NET. Our results are comparable to those found by Pabst et al. [(16); *g* = 0.7] and also in line with recent results of a study that brief intensive trauma-focused treatment for PTSD significantly decreased Borderline symptom severity [Cohen's *d* = 0.7; (43)].

DBT-bt performed better than expected in terms of trauma symptom severity (CAPS: *g* = 0.8, PDS: *g* = 0.7). Mixed linear models did not indicate a more pronounced decrease of PTSD symptom severity in NET. At a first sight, these results are contrary to studies showing that DBT is insufficient in treating PTSD (6). However, only 6.2% of patients in DBT-bt remitted from PTSD at 12-months follow-up. The remission rate was significantly higher in patients who received NET (33.3%, intention-to-treat sample: 32.0%). Notably, irrespective of treatment condition all patients who had PTSD remission also recovered from BPD. This was not the case vice versa. Only 44.4% of patients who remitted from BPD also recovered from PTSD and PTSD persisted in 90% patients who remitted from BPD after DBT-bt. Despite the large PTSD effect sizes in DBT-bt, this suggests that trauma-focused therapy is necessary to recover from PTSD and potentially changes other comorbid conditions (10). By that, like a number of studies now, our study speaks to the value of trauma-focused psychotherapy in this patient population. Also, it highlights the risk that PTSD may become chronic without being treated specifically and may complicate treatment of BPD (44). Remission rates in our study (33%, ITT: 32%) are overall comparable with the results by Bohus et al. (8). DBT-PTSD showed a PTSD response rate of 29.4% (vs. 40% in our study) and a remission rate of 41.2% in the subsample of PTSD patients meeting criteria for BPD (vs. 33.3% in our study).

Even though around 50% of patients showed reliable improvements in PTSD and BPD pathology after NET, a small amount of patients in NET got reliably worse until 12-months follow-up, which was not true in DBT-bt and other studies evaluating trauma-focused treatment in this highly burdened sample. This may argue for trauma-focused practices to be integrated into DBT, as is the case with DBT-PTSD. However, reliable worsening in our study did not occur until 12-months follow-up. As studies evaluating DBT-PTSD used a much shorter follow-up interval, it remains unclear whether there are similar courses in other treatment programs. Notably, the differences in reliable worsening in our study did not differ between treatment groups. It explicitly cannot be concluded from our data that NET or trauma-focused procedures in general are unsafe.

Higher remission rates as well as reliable worsening in NET suggested that there was a large interindividual variance in PTSD improvement which was confirmed by a significantly higher variance at 12-months follow-up compared to DBT-bt. The difference in variance may explain why the superiority of NET in remission rates did not emerge in the mixed linear models. Taken together, a proportion of patients benefited greatly from NET, another did not or got even worsen in the follow-up period. It is important to find out which factors predict stable treatment success.

The value of trauma-focused treatment in this patient group also becomes apparent in the dropout rates found in this study, which was significantly lower in NET. The high dropout rate in DBT-bt needs to be interpreted with caution, because DBT-bt is the standard treatment in our ward and the dropout rate is usually lower on our ward (45). NET was only available within the study conditions and it is nearly impossible for our seriously ill patients to access outpatient trauma-focused treatment. This means that only patients participated in the study who hoped for a trauma-focused treatment or assumed they could benefit from both treatments (otherwise they would have chosen the standard DBT-bt program). The higher dropout rate may therefore also illustrate the great need for trauma-focused treatment for this patient group and disappointment in those who did not receive it immediately. This effect may also explain the elevated depression scores in DBT-bt on admission.

Several limitations of this study need to be considered. Unfortunately, the German versions of the DSM-5 instruments were introduced shortly after the study began, therefore, our PTSD-measures are now outdated. Furthermore, we did not use a specific diagnostic interview for BPD such as the Zanarini Rating Scale. However, the total number of BPD criteria is an adequate approximation of BPD severity (46). We do not have a specialized study infrastructure that allows treatment of the same patients in different wards with separated treatment concepts. Therefore, it is likely that both patient groups interacted with each other. NET patients may have learned skills this way and DBT-bt patients may have realized that it is helpful to reduce avoiding trauma-associated emotions. Outpatient psychotherapy studies are much better suited to disentangle the effects of individual therapy programs. However, as residential treatment programs are common in the EU and psychotherapists still have safety concerns with regard to outpatient trauma-focused treatment (47), it is important to evaluate residential treatment programs that allow patients to get the treatment they need. To avoid biased results we included all randomized participants, even if they left the ward within a few days, and ran intention-to-treat analyses. Nevertheless, an attrition bias cannot be ruled out. The correct execution of therapies was only ensured by supervision, we did not conduct adherence rating. We also could not consistently ensure that clinical raters were blinded after randomization. The long follow-up interval is both a strength and weakness of this study. Many factors can affect the further course of a treatment. We considered biasing factors; however, e.g., quantity and quality of treatment in the interim can still only be estimated. Future research should address the potential socioeconomic benefits of trauma-focused procedures in BPD.

## Conclusions

The study shows that NET was effective in reducing PTSD symptom severity showing large effect sizes and leading to significantly higher PTSD remission rates and lower dropout rates than DBT-bt in a residential setting. Remission in PTSD was in all cases accompanied by remission in BPD highlighting the value of trauma-focused in this highly burdened patient group for recovery in both disorders. To shorten the duration of both illnesses as much as possible, future studies should focus on the factors predicting treatment success and enabling patients to benefit from trauma-focused treatment.

## Data Availability Statement

The datasets presented in this article are not readily available in order to preserve the anonymity of participating patients. Requests to access aggregated data should be directed to Carolin Steuwe, carolin.steuwe@evkb.de.

## Ethics Statement

This study was reviewed and approved by the Ethics Committee of the Medical Association Westfalen-Lippe and the University of Münster. All patients/participants provided their written informed consent to participate in this study.

## Author Contributions

CS recruited patients, conducted therapies, organized and supervised the post-treatment assessments, and performed the statistical analysis and drafted the manuscript. MB participated in study design and manuscript preparation and supervised therapies. TB participated in study design and manuscript preparation. MD participated in study design and manuscript preparation. All authors read and approved the final manuscript.

## Funding

We acknowledge support for the publication costs by the Open Access Publication Fund of Bielefeld University.

## Conflict of Interest

The authors declare that the research was conducted in the absence of any commercial or financial relationships that could be construed as a potential conflict of interest.

## Publisher's Note

All claims expressed in this article are solely those of the authors and do not necessarily represent those of their affiliated organizations, or those of the publisher, the editors and the reviewers. Any product that may be evaluated in this article, or claim that may be made by its manufacturer, is not guaranteed or endorsed by the publisher.
