# Supplementary material for: Narrative Exposure Therapy in Patients With Posttraumatic Stress Disorder and Borderline Personality Disorder in a Naturalistic Residential Setting: A Randomized Controlled Trial
Source: Front Psychiatry. 2021 Nov 26;12:765348. doi: 10.3389/fpsyt.2021.765348 (PMC8663140; doi:10.3389/fpsyt.2021.765348)
Supplement: Supplementary file 1 [file Data_Sheet_1.docx]

**Supplementary Material**

*Standard Inpatient Care*

All patients received non-specific therapeutic elements that were identical for both intervention arms. Participants received twice-weekly 30 min sessions of supportive talks with the primary nurse (additional contacts in crisis situations were documented), twice-weekly sessions of art- or music therapy (randomized), and weekly sessions of body therapy. Beyond that all patients received morning meetings, movement therapy, and learned relaxation techniques. Patients received usual psychopharmacological treatment that was documented. Irrespective of group membership, no benzodiazepines and no on-demand medication were used.

*Treatment dose*

It was difficult to parallelize the treatment dose as NET takes place in individual therapy sessions whereas DBT combines individual therapy with group therapy. The total therapy dose in NET was 1,330 minutes of individual therapy. In DBT 500 minutes of individual therapy were conducted as well as a total of 2850 minutes of group therapy. Here we considered 200 minutes of group therapy to be equivalent to 50 minutes of individual therapy. Therefore 2,850 hours of group therapy in 10 weeks of treatment correspond to 712.5 minutes of “individual” group therapy in DBT-bt resulting in a total therapy dose of 1,212.5 minutes in DBT-bt and 1,330 minutes in NET. Patient groups did not differ in the number of treatment days, *t*(38)=1.30, p=.201.

*Therapists*

Therapists (*n*=10) were primarily female (80%), held a master’s degree in psychology (80%) and had a mean of 1.4 (*SD*=1.52, *median*=0.5) years of clinical experience. All therapists had been trained in DBT and NET. Supervision was given once a week by a licensed professional trained in both DBT and NET. The therapists were equally distributed across treatment conditions, *χ*^2^(9)=12.95, *p*=.165, and working experience of therapists in both conditions did not differ, *z*=-0.90, *p*=.367.

Table S1

*Observed cases for all primary and secondary outcome measures*

|  | Treatment completer sample  (*n*=40) | |  | Intention-to-treat sample  (*n*=58) | |  |
| --- | --- | --- | --- | --- | --- | --- |
|  | NET | DBT-bt |  | NET | DBT-bt |  |
| CAPS |  |  |  |  |  |  |
| Pre-treatment (*n*) | 23^a^ | 16 |  | 28^a^ | 29 |  |
| Post-treatment (*n*) | 22 | 16 |  | 24 | 19 |  |
| 12-months follow-up (*n*) | 21 | 16 |  | 25 | 22 |  |
| SCID-II-BPD |  |  |  |  |  |  |
| Pre-treatment (*n*) | 24 | 16 |  | 29 | 29 |  |
| Post-treatment (*n*) | - | - |  | - | - |  |
| 12-months follow-up (*n*) | 21 | 16 |  | 25 | 22 |  |
| PDS |  |  |  |  |  |  |
| Pre-treatment (*n*) | 24 | 16 |  | 28 | 23 |  |
| Post-treatment (*n*) | 24 | 16 |  | 26 | 19 |  |
| 12-months follow-up (*n*) | 16 | 15 |  | 20 | 21 |  |
| BLS |  |  |  |  |  |  |
| Pre-treatment (*n*) | 24 | 16 |  | 28 | 23 |  |
| Post-treatment (*n*) | 24 | 16 |  | 26 | 19 |  |
| 12-months follow-up (*n*) | 16 | 15 |  | 20 | 21 |  |
| BDI |  |  |  |  |  |  |
| Pre-treatment (*n*) | 23 | 16 |  | 27 | 23 |  |
| Post-treatment (*n*) | 24 | 16 |  | 26 | 19 |  |
| 12-months follow-up (*n*) | 16 | 15 |  | 20 | 21 |  |
| BDI |  |  |  |  |  |  |
| Pre-treatment (*n*) | 23 | 16 |  | 27 | 23 |  |
| Post-treatment (*n*) | 24 | 16 |  | 26 | 19 |  |
| 12-months follow-up (*n*) | 16 | 15 |  | 20 | 21 |  |
| DES |  |  |  |  |  |  |
| Pre-treatment (*n*) | 23 | 16 |  | 27 | 24 |  |
| Post-treatment (*n*) | 24 | 16 |  | 26 | 19 |  |
| 12-months follow-up (*n*) | 16 | 15 |  | 20 | 20 |  |
| SCL-90-R |  |  |  |  |  |  |
| Pre-treatment (*n*) | 24 | 16 |  | 28 | 23 |  |
| Post-treatment (*n*) | 24 | 15 |  | 26 | 18 |  |
| 12-months follow-up (*n*) | 16 | 15 |  | 20 | 20 |  |
| WHOQOL |  |  |  |  |  |  |
| Pre-treatment (*n*) | 22 | 16 |  | 25 | 23 |  |
| Post-treatment (*n*) | 24 | 16 |  | 26 | 19 |  |
| 12-months follow-up (*n*) | 16 | 15 |  | 20 | 20 |  |

**Note.** NET = Narrative Exposure Therapy; DBT-bt = Dialectical Behavior Therapy (DBT) based treatment; CAPS = Clinician Administered PTSD Scale (Blake, 2000); SCID-II = Structured Clinical Interview for DSM-IV, Personality Disorders; BPD = Borderline Personality Disorder; PDS = Posttraumatic Stress Diagnostic Scale; BSL = Borderline Symptom List; BDI-II = Beck Depression Inventory Revision. DES = Dissociative Experience Scale; SCL-90-R = Symptom Checklist 90 Items Revised Version; WHOQOL = World Health Organisation Quality of Life. ^a­^ The total score of the CAPS at T1 was missing for one participant due to an incomplete assessment.

Table S2

*Reasons for treatment drop-out*

| Reasons for droptout | NET | DBT-bt |
| --- | --- | --- |
| 1 | Could not cope with the residential setting, wanted to stay overnight with her partner (within the first week) | Overload and repeated uncontrollable self-injury (within the first two weeks) |
| 2 | Repeated devaluations of staff and damage to property | Repeated acute suicidality and transfer to the acute psychiatric ward, non-compliance with ward rules |
| 3 | Repeated alcohol consumption | Repeated alcohol consumption and self-injury |
| 4 | Wish to end treatment, fear of trauma therapy (discharge before exposure started) | Lack of commitment to the use of tension-regulating skills and no realistic goals for the treatment (within the first week) |
| 5 | Repeated partnership conflicts, not returned from a temporary leave | Repeated insults and devaluations of the therapeutic staff |
| 6 |  | Repeated alcohol consumption |
| 7 |  | Could not cope with the residential treatment (within the first week) |
| 8 |  | Partnership conflicts, alcohol consumption |
| 9 |  | Repeated acute suicidality and transfer to the acute psychiatric ward, non-compliance with ward rules |
| 10 |  | Repeated massive self-injuries and transfer to the acute psychiatric ward, two suicide attempts there |
| 11 |  | Did not feel “ready” for DBT (within the first week) |
| 12 |  | Repeated transfers to the acute psychiatric ward, violation of ward rules |
| 13 |  | No commitment to work on the diary card, acute illness of the father-in-law, did not appear for continuation of treatment |

**Note.** NET = Narrative Exposure Therapy; DBT-bt = Dialectical-Behavior Therapy (DBT) based treatment. Gray shading indicates discharge on part of the patient, white shading indicates discharge on part of the ward.

Table S3

*Critical life events and outpatient/residential treatment in the follow-up period.*

|  | Treatment completer sample | |  | Intention-to-treat sample | |  |
| --- | --- | --- | --- | --- | --- | --- |
|  | NET | DBT-bt |  | NET | DBT-bt |  |
| Traumatic life events (%) | 10% | 0% |  | 8% | 10% |  |
| *p* values | .204 | |  | .848 | |  |
| Critical life events (%) | 62% | 63% |  | 67% | 67% |  |
| *p* values | .970 | |  | 1.000 | |  |
| Outpatient psychotherapy (%) | 55% | 69% |  | 56% | 64% |  |
| *p* values | .376 | |  | .595 | |  |
| Outpatient DBT skills group (%) | 18% | 13% |  | 16% | 9% |  |
| *p* values | .635 | |  | .479 | |  |
| Outpatient psychotherapy (DBT based, %) | 0% | 0% |  | 0% | 5% |  |
| *p* values | - | |  | .270 | |  |
| Outpatient psychotherapy (trauma-focused, %) | 5% | 13% |  | 12% | 9% |  |
| *p* values | .369 | |  | .747 | |  |
| Inpatient/residential treatment (%) | 32% | 44% |  | 36% | 45% |  |
| *p* values | .640 | |  | .433 | |  |
| Inpatient/residential treatment in days (*M*, *SD*) | 7.52 (15.84) | 37.88 (82.31) |  | 14.58 (33.21) | 32.27 (71.17) |  |
| *p* values | .253^a^ | |  | .362^a^ | |  |
| Residential treatment (DBT based, %) | 0% | 0% |  | 4% | 0% |  |
| *p* values | - | |  | .343 | |  |
| Residential treatment (trauma-focused, %) | 5% | 0% |  | 4% | 0% |  |
| *p* values | .376 | |  | .333 | |  |
| Crisis intervention (%) | 32% | 37% |  | 32% | 36% |  |
| *p* values | .820 | |  | .745 | |  |
| Crisis intervention in days (*M*, *SD*) | 5.81 (13.99) | 35.25 (82.84) |  | 6.83 (15.13) | 29.73 (71.67) |  |
| *p* values | .440^a^ | |  | .461^a^ | |  |

**Note.** NET = Narrative Exposure Therapy; DBT-bt = Dialectical Behavior Therapy (DBT) based treatment. Data are available only for patients who were observed at 12-months follow-up. All variables were coded as dichotomous variables (“at least one”), except for the number inpatient/residential treatment days. Dichotomous variables were compared using χ^2^ tests, and marked variables (^a^) were compared using Mann-Whitney U tests.

Table S4

*Results of mixed-effects models for secondary outcomes in patients with sexual abuse.*

|  | Treatment completer sample (*n* = ) | | | | | | | | |  | | Intent-to-treat sample (*n* = ) | | | | | | |  |
| --- | --- | --- | --- | --- | --- | --- | --- | --- | --- | --- | --- | --- | --- | --- | --- | --- | --- | --- | --- |
|  | Main effects | | | | |  | | Interaction | |  | | Main effects | | | |  | | Interaction |  |
|  | Time | | | Treatment | |  | | Time x treatment | |  | | Time | | Treatment | |  | | Time x treatment |  |
| CAPS | 18.49_2,51_*** | | | 0.47_1,28_ | |  | | 0.21_2,51_ | |  | | 19.58_2,62_*** | | 1.88_1,37_ | |  | | 0.14_2,62_ |  |
| SCID-II-BPD | 22.10_1,27_*** | | | 0.66_1,29_ | |  | | 0.14_1,27_ | |  | | 33.70_1,34_*** | | <0.01_1,38_ | |  | | <0.01_1,34_ |  |
| PDS | | 11.74_2,49_*** | 1.87_1,28_ | |  | | 0.50_2,49_ | |  | | 20.59_2,60_*** | | 4.15_1,37_* | |  | | 0.12_2,60_ | |  |
| SCID-II-BPD | 22.10_1,27_*** | | | 0.66_1,29_ | |  | | 0.14_1,27_ | |  | | 33.70_1,34_*** | | <0.01_1,38_ | |  | | <0.01_1,34_ |  |
| BSL | | 12.37_2,49_*** | 0.93_1,28_ | |  | | 1.68_2,49_ | |  | | 16.87_2,60_*** | | 1.76_1,37_ | |  | | 0.61_2,60_ | |  |
| BDI-II | 25.70_2,53_*** | | | 3.73_1,31_ | |  | | 2.05_2,53_ | |  | | 25.79_2,64_*** | | 5.48_1,43_* | |  | | 0.67_2,64_ |  |
| DES | 8.03_2,48_** | | | 1.30_1,28_ | |  | | 1.77_2,48_ | |  | | 10.57_2,58_*** | | 1.53_1,37_ | |  | | 1.53_2,58_ |  |
| SCL-90-R | 12.45_2,48_*** | | | 2.60_1,28_ | |  | | 0.55_2,48_ | |  | | 16.66_2,58_ | | 2.31_1,37_ | |  | | 0.11_2,58_ |  |
| WHOQOL | 4.38_2,47_* | | | 0.11_1,28_ | |  | | 1.85_2,47_ | |  | | 6.91_2,56_** | | 1.18_1,37_ | |  | | 1.23_2,56_ |  |

**Note.** All results are presented as F-values (F_d1,d2_). Baseline depression was entered as a fixed factor in all models except for the depression models. NET = Narrative Exposure Therapy; DBT-bt = Dialectical-Behavior Therapy; CAPS = Clinician Administered PTSD Scale (Blake, 2000); PDS = Posttraumatic Stress Diagnostic Scale; SCID-II = Structured Clinical Interview for DSM-IV, Personality Disorders; BPD = Borderline Personality Disorder; BSL = Borderline Symptom List; BDI-II = Beck Depression Inventory Revision. DES = Dissociative Experience Scale; SCL-90-R = Symptom Checklist 90 Items Revised Version; WHOQOL = World Health Organisation Quality of Life (higher values indicate more quality of life).

**p* ≤.05. ***p* ≤.01. ****p* ≤.001.


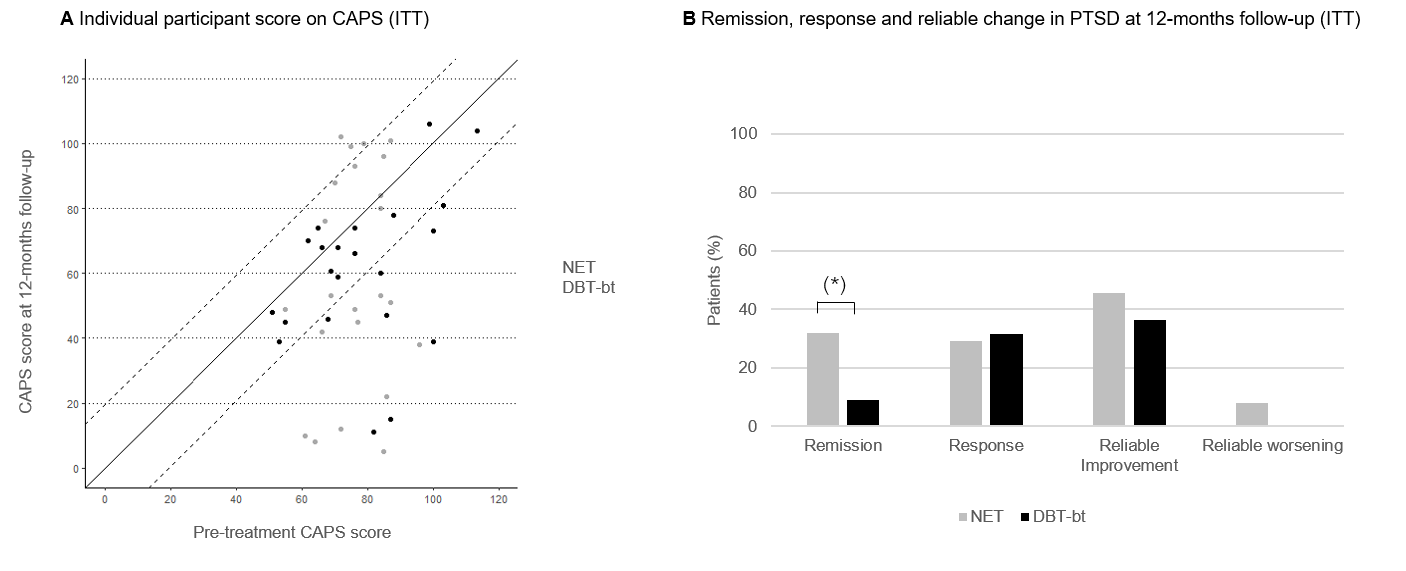


*Fig. S2.* **A** Total Clinician-Administered PTSD Scale for DSM-IV (CAPS; Blake, 2000) scores of participants randomized to Narrative Exposure Therapy (NET) or Dialectical Behavior Therapy (DBT) based Treatment (DBT-bt) before therapy and at 12-months follow-up. Values below the main diagonal indicate improvements, the dotted diagonals show reliable change. **B** Categorial treatment outcomes for the CAPS at 12-months follow-up. Data are presented for the intention-to-treat sample, (*)*p* =.053.


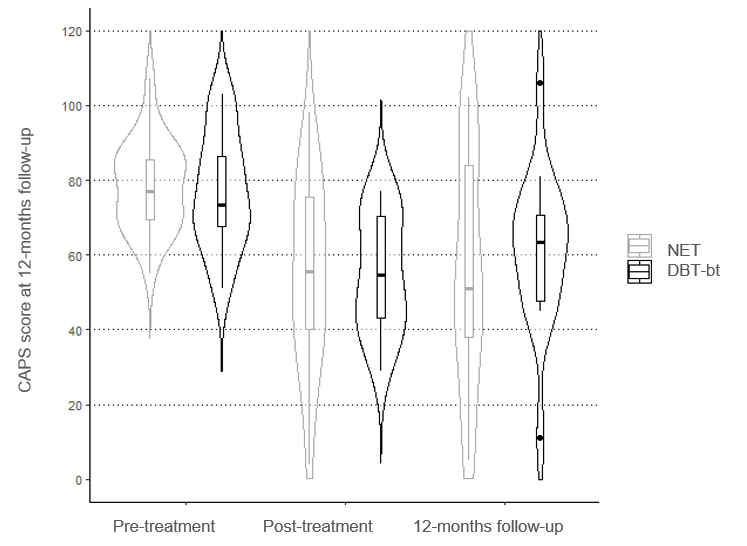


*Fig. S3a.* Scores and distribution based on the Clinician-Administered PTSD Scale (CAPS; Blake, 2000) for treatment completers of Narrative Exposure Therapy (NET; grey colour) and Dialectical Behavior Therapy (DBT) based Treatment (DBT-bt, black colour). Boxplots indicate means and standard deviations (dots show outliers). Violin plots show the different distribution of CAPS scores for each treatment group. Data are presented for the treatment completer sample, for the intention-to-treat sample, see Figure S3b.


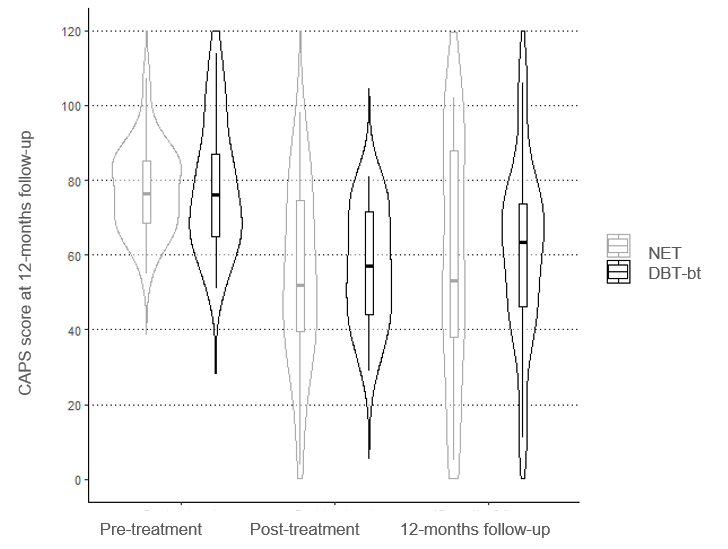


*Fig. S3b.* Scores and distribution of the intention-to-treat sample based on the Clinician-Administered PTSD Scale (Blake, 2000) for Narrative Exposure Therapy (NET; grey colour) and Dialectical Behavior Therapy (DBT) based Treatment (DBT-bt, black colour). Boxplots indicate means and standard deviations (dots show outliers). Violin plots show the different distribution of CAPS scores for each treatment group.
